# The Impact of Action Effects on Infants’ Predictive Gaze Shifts for a Non-Human Grasping Action at 7, 11, and 18 Months

**DOI:** 10.3389/fpsyg.2021.695550

**Published:** 2021-08-10

**Authors:** Maurits Adam, Christian Gumbsch, Martin V. Butz, Birgit Elsner

**Affiliations:** ^1^Developmental Psychology, Department of Psychology, University of Potsdam, Potsdam, Germany; ^2^Neuro-Cognitive Modeling, Department of Computer Science and Department of Psychology, University of Tübingen, Tübingen, Germany; ^3^Autonomous Learning Group, Max Planck Institute for Intelligent Systems, Stuttgart, Germany

**Keywords:** infancy, predictive gaze behavior, eye tracking, tool-use actions, agency cues, developing agentive self, non-human grasping

## Abstract

During the observation of goal-directed actions, infants usually predict the goal at an earlier age when the agent is familiar (e.g., human hand) compared to unfamiliar (e.g., mechanical claw). These findings implicate a crucial role of the developing agentive self for infants’ processing of others’ action goals. Recent theoretical accounts suggest that predictive gaze behavior relies on an interplay between infants’ agentive experience (top-down processes) and perceptual information about the agent and the action-event (bottom-up information; e.g., agency cues). The present study examined 7-, 11-, and 18-month-old infants’ predictive gaze behavior for a grasping action performed by an unfamiliar tool, depending on infants’ age-related action knowledge about tool-use and the display of the agency cue of producing a salient action effect. The results are in line with the notion of a systematic interplay between experience-based top-down processes and cue-based bottom-up information: Regardless of the salient action effect, predictive gaze shifts did not occur in the 7-month-olds (least experienced age group), but did occur in the 18-month-olds (most experienced age group). In the 11-month-olds, however, predictive gaze shifts occurred only when a salient action effect was presented. This sheds new light on how the developing agentive self, in interplay with available agency cues, supports infants’ action-goal prediction also for observed tool-use actions.

## Introduction

Humans live in a world that is filled with goal-directed actions: People grasp for objects, use tools for crafting, or extend their hands toward each other to shake them. Per definition, an action is a movement that is performed by an agent in order to obtain a desired goal (Prinz, 1997). Sometimes, it can be crucial to predict the goal of the observed action in order to react accordingly and in a timely manner. In a social environment, this goal prediction is important both in the context of competitive and cooperative situations, which is why its development has been studied extensively over the past decades. Because infants’ ability for action prediction is related to their emerging action experience, it is crucial to ask how the developing agentive self supports the processing of non-human action goals.

In developmental psychology, a common measure for infants’ ability for action prediction is predictive gaze shifts (e.g., Falck-Ytter et al., 2006; Kanakogi and Itakura, 2011; Ambrosini et al., 2013; Adam et al., 2017). For example, when an infant observes how an agent approaches and grasps a goal object, a predictive gaze shift is coded when the infant’s gaze moves from the moving agent to the goal object before the agent arrives there. This predictive gaze behavior has been proposed to reflect attentional mechanisms, where the overt shift of the gaze position from the moving agent to the goal object is preceded by covert shifts of attention to the goal object, given that an agent has been detected (Deubel and Schneider, 1996; Daum and Gredebäck, 2010; Gredebäck and Daum, 2016). Therefore, predictive gaze behavior is a suitable means to investigate infants’ ability to identify agents and to process actions as being directed toward a goal.

Starting around 6 to 7 months of age, infants show goal-predictive gaze behavior when observing simple human grasping actions; that is, they shift their gaze to the to-be-reached goal object before the agent arrives at its goal (e.g., Kanakogi and Itakura, 2011; Ambrosini et al., 2013; Adam and Elsner, 2020). A little later, at around 12 months of age, infants even predict the goals of more complex human actions, such as transporting toys into a bucket (Falck-Ytter et al., 2006). It is often suggested that infants’ goal-predictive gaze behavior is closely linked to the infants’ developing agentive self, acquiring sensorimotor experience with all kinds of actions and their consequences, and infants’ ability to perform the observed actions themselves (e.g., Falck-Ytter et al., 2006; Kanakogi and Itakura, 2011; Melzer et al., 2012). This is evidenced by correlations between infants’ abilities to perform certain actions and their ability to predict the goal of these actions, as well as by studies showing that infants struggle to predict the goal of actions performed by non-human agents or of actions they are not yet able to perform themselves (e.g., Falck-Ytter et al., 2006; Kanakogi and Itakura, 2011; Cannon and Woodward, 2012; Adam et al., 2017). Additional support comes from looking-time research, in which infants’ attribution of goal-directedness to an observed action was measured *post-hoc*, that is, after the action goal had been completed. For example, from 6 months on, infants attribute goals to grasping actions by human hands, but not when a hand touches a goal object with its back, which is an unfamiliar action, or when the grasping action is performed by a mechanical claw, which is an unfamiliar agent (e.g., Woodward, 1998, 1999). Furthermore, at 3 months of age, infants’ own production of actions was reported to have a larger impact on infants’ goal attribution than have simple observations of the same actions without own production (Gerson and Woodward, 2014a). This suggests that own agentive experience is especially crucial for subsequent action processing, which is also supported by computational models and social developmental data (e.g., Pavlova, 2012; Butz and Kutter, 2017).

The aim of the present study was to investigate 7-, 11-, and 18-month-old infants’ predictive gaze behavior during the observation of simple grasping actions performed by an unfamiliar mechanical claw. We expected that predictive gaze behavior will develop across the age groups, presumably due to the infants’ increasing prior knowledge about the observed action from own sensorimotor experience and from observing others. Additionally, we studied whether the production of a salient action effect, as a potential agency cue (e.g., Bíró and Leslie, 2007), influences infants’ predictive gaze behavior. Recent research suggests that infants are able to predict the goal of actions by non-human agents, as long as these agents exhibit certain behavioral agency cues, such as self-propelled movement, equifinality of goal achievement, or the ability to produce salient action effects (e.g., Bíró, 2013; Adam and Elsner, 2018). For example, Adam and Elsner (2018) presented 11-month-olds with videos of a mechanical claw approaching a toy on a linear path. Remarkably, infants showed goal-predictive gaze shifts not only when the claw showed all three agency cues but also when the claw just grasped the toy and lifted it, therefore displaying only one agency cue, that is, the salient action effect of lifting the toy, which was additionally marked by a sound. This suggests that the action effect was especially important for the infants to predict the observed agent’s goal (see Bíró et al., 2014, for similar findings regarding the importance of action effects). In another condition, the claw just grasped the toy and then froze in place. In this case, infants showed tracking gaze behavior; that is, they looked at the claw until it reached the goal. These results are in line with ideomotor accounts proposing that actions are primarily represented by the effects they elicit, which highlights the crucial role action effects play for infants’ ability to predict the goal of an observed action event (e.g., Prinz, 1997; Elsner and Hommel, 2001).

Gumbsch et al. (2021) developed a generative, event-predictive computational model that successfully modeled the development of infants’ gaze behavior when observing human and non-human agents performing goal-directed actions. The Cognitive Action Prediction Model in Infants (CAPRI) proposes that infants generate internal probabilistic generative models of observed action events and transitions between events. These internal models are suggested to develop through infants’ sensorimotor interaction with the environment (e.g., when infants repeatedly grasp for and interact with objects or observe others doing so). Based on the free energy minimization formalism (Friston, 2010; Friston et al., 2015), during action generation and observation, CAPRI actively infers gaze behavior *via* the objective to minimize uncertainty about the probabilistically inferred ongoing and upcoming interactions. Critically, the involved learned, generative, and event-predictive models (Zacks et al., 2007; Butz, 2016; Butz et al., 2021) segment the continuous sensorimotor experiences into event and event-transition encodings, thus enabling deeper considerations about the upcoming events. As a result, predictive gaze behavior developed when CAPRI was trained on object interaction events – in this case not considering differences between observing or executing actions. With hardly any knowledge about grasping actions, the model tracked the moving hand to minimize uncertainty and to gain information about its future position. While learning from the accumulating experience with grasping events, predictive gaze shifts developed, because CAPRI aims at minimizing the uncertainty about whether, when, and how the hand is going to grasp the target object (Gumbsch et al., 2021).

Similarly, Elsner and Adam (2020) argued that actions can be seen as events that are cognitively stored as feature bundles (Hommel et al., 2001; Zacks et al., 2007; Butz, 2016). *Via* an interaction between bottom-up and top-down processes, infants’ generation of predictive gaze behavior is suggested to be based on three essential steps. First, bottom-up features of the ongoing, yet incomplete, action have to be perceived and processed. These features include, for example, the agent‘s appearance or the kinematics of the movement toward the goal. Second, this bottom-up information is mapped onto stored action-event schemata, that is, cognitive action representations (Elsner and Hommel, 2001; Zacks et al., 2007; Butz, 2016). Generally, schemata represent organized knowledge that describes different concepts, such as situations or events (Schützwohl, 1998). Therefore, an action-event schema, for example, encodes information acquired through experience as an agentive self, in the form of sensorimotor feature nodes connected by associations of various strength. Schemata are typically used to properly comprehend current input or to predict future input, and therefore, schemata are constantly tested against their compatibility with the observed situation (Schützwohl, 1998). Consequently, the associative network underlying a specific schema is updated frequently, and previously learned associations are adjusted based on new learning experiences. In the case of action-event schemata, the number of feature nodes and the strength of the associations increase upon each performance or observation of an action. For example, action-event schemata encode that when a hand moves toward an object, typically a salient action effect follows once the hand closes-in on that object. Third, when sufficient action experience is available, the perception of bottom-up information about the agent, potential goal object, and start of the movement triggers the inference of a “reaching” action-event schema. This then routes the anticipation of an upcoming salient action effect upon reaching the object, which leads to predictive gaze behavior because the active inference process strives to decrease anticipated effect uncertainty (Elsner and Adam, 2020; Gumbsch et al., 2021).

According to these model considerations, as long as infants have only little to no experience with an observed action or agent, they should not be able to predict the action goal. Instead, tracking the unfolding bottom-up information helps infants to understand the ongoing action, thereby adding feature nodes and strengthening the associations between them in the developing action-event schema. Infants normally gather experience about agents that display various agency cues (e.g., Bíró and Leslie, 2007; Bíró, 2013), and this perceivable bottom-up information appears to be stored in action-event schemata. With accumulating experience, the mere perception of the agent’s features or the initial state of the action event becomes sufficient to activate the action-event schema, enabling successful goal predictions (e.g., Elsner and Adam, 2020). Following this idea, when unfamiliar agents, such as mechanical claws, display one or more agency cues, corresponding event schemata (linked to the agency cue) can become activated. As a result, the unfamiliar agent or its observable features, such as its appearance, may become associated with the event schemata. In subsequent trials, these top-down influences then allow for predictive gaze behavior even for the unfamiliar agent.

This is in line with looking-time research showing that infants at 6 months need to see more agency cues than older infants at 9 or 12 months in order to attribute a goal to the action of a mechanical claw (Bíró and Leslie, 2007). Moreover, when 9-month-olds were presented with a situation that suggested that a mechanical claw was about to act goal-directedly, infants’ EEG response showed patterns of goal identification (Southgate and Begus, 2013). Finally, in eye-tracking studies, adults showed goal-predictive gaze shifts for unusual hand actions or for grasping by a mechanical claw, even in the absence of any additional agency cues (Kanakogi and Itakura, 2011; Adam et al., 2017). Taken together, these results illustrate how observers with limited knowledge about the observed action rely on the unfolding bottom-up information, whereas observers with more knowledge can rely on their stored top-down information that they have gathered through prior knowledge or experience with an action event or an agent.

A recent study investigated infants’ use of bottom-up- versus top-down information across the first year of life, by repeatedly presenting 6-, 7-, and 11-month-olds with a hand that approached and grasped a goal object, followed either by a salient action effect (e.g., lifting up the object, accompanied by a sound) or by just freezing in place (Adam and Elsner, 2020). At 6 months, infants showed tracking gaze behavior regardless of the salient action effect, confirming the assumed behavior of infants who have just recently accomplished the motor development milestone of visually guided grasping. In contrast, at 11 months, when infants are experienced in grasping, predictive gaze behavior occurred in both conditions. Interestingly, at 7 months, infants were predictive in the human-hand condition only with the salient action effect and did not show predictive gaze behavior when a grasping mechanical claw produced the salient action effect. These results might reflect that 7-month-olds’ representations for human grasping actions are still weak and were only activated *via* additional agency cues and when infants observed a human hand. For the claw, however, the 7-month-olds did not yet conceive of action representations that could be activated, and therefore, the agency cue did not lead to predictive gaze behavior. These results highlighted how the developing agentive self in infancy might help to shape infants’ ability to predict the goals of observed action events.

The current study aimed at taking this idea a step further and at investigating the assumed role of the developing agentive self and the interplay of top-down and bottom-up information in the context of predictive gaze behavior for a non-human grasping action. If observers, depending on their knowledge about an observed action, indeed rely on either prior top-down knowledge about the observed action or on presented bottom-up information when generating predictive eye movements, we should see similar developmental patterns, albeit at different ages, for familiar agents and for unfamiliar agents. For example, compared to human hands, infants have much less conceptual knowledge about how mechanical claws are able to grasp and manipulate objects, or about how claws can be used as tools. Therefore, we predicted that the results by Adam and Elsner (2020) across different age groups could be replicated with an unfamiliar agent such as a mechanical claw, when the infants are older and have more experience with grasping in general, but also with the use of tools (e.g., McCarty et al., 2001). Additionally, there should be an age at which infants possess sufficient action knowledge and would predict the goal of a mechanical claw even without any additional agency cues.

Therefore, we recorded 7-, 11-, and 18-month-olds’ eye movements, while infants repeatedly watched a video in which a mechanical claw approached and grasped a goal object and then either did or did not produce a salient action effect. We investigated first, at which age infants would use the agency cue to predict the goal of a simple grasping action performed by a mechanical claw. Second, we investigated whether there would be a learning process that manifests itself as faster gaze shifts to the goal across trials. The three age groups were chosen based on prior research: We expected the 7-month-olds to not show predictive gaze shifts regardless of the salient action effect based on 7-month-olds’ limited knowledge about both grasping actions and tool-use actions, and on research reporting that 7-month-olds do not predict the goal of a mechanical claw even when it produces salient action effects (McCarty et al., 2001; Adam and Elsner, 2020). We expected the 11-month-olds to be predictive in the condition with the action effect, but to show tracking gaze behavior in the condition without the action effect, because they have more knowledge about grasping actions than the 7-month-olds. In previous studies, 11-month-olds, who still have relatively limited experience with tool-use actions (McCarty et al., 2001), showed predictive gaze behavior when a grasping mechanical claw produced a salient action effect, but tracked a mechanical claw in the absence of additional agency cues (Adam and Elsner, 2018). Finally, we expected the 18-month-olds to show predictive gaze behavior regardless of the action effect, because infants at that age should already have sufficient knowledge about grasping actions and tool use. Specifically, between 14 and 19 months, infants start to engage in successful actions with claw-like tools to obtain distant objects (McCarty et al., 2001). Therefore, at 18 months, the advanced agentive self should enable goal-prediction *via* top-down processes upon perceiving the start of the claw’s grasping, even without agency cues.

## Materials and Methods

### Participants

The final sample consisted of forty-two 7-month-olds (*M =* 6.9, *SD =* 0.3, range = 6.5–7.5 months, 20 girls), forty-one 11-month-olds (*M =* 10.9, *SD =* 0.3, range = 10.5–11.5 months, 21 girls), and forty-one 18-month-olds (*M =* 18.0, *SD =* 0.3, range = 17.5–18.6 months, 22 girls). An additional 9, 8, and 4 participants, respectively, were tested but had to be excluded because they did not contribute enough valid data (criteria see below). The participants were randomly assigned to either the action-effect condition (7-month-olds: *n =* 21; 11-month-olds: *n =* 20; and 18-month-olds: *n =* 19) or the no-action-effect condition (7-month-olds: *n =* 21; 11-month-olds: *n =* 21; and 18-month-olds: *n =* 22). The parents and their children were recruited from a database where parents can sign up their child to participate in studies in the babylab. Participants mostly came from middle-class families in a small German city. During their stay at the laboratory, parents signed informed consent and received 7.50 € as well as a certificate with a photograph of their child as reimbursement. This study was approved by the Ethics Committee of the University of Potsdam.

### Stimuli, Apparatus, and Procedure

**Figure 1:**
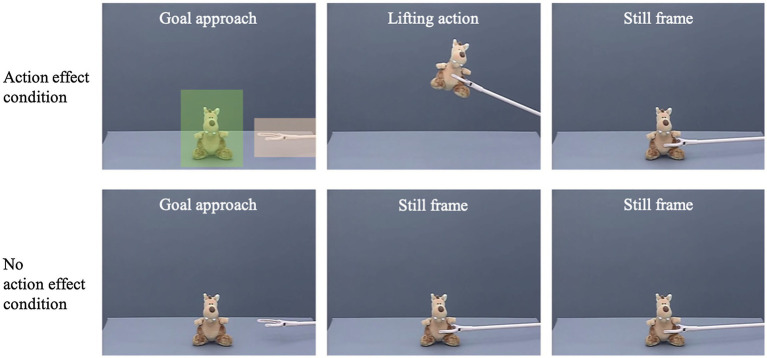
Still frames of the stimulus videos in the action-effect condition (upper row) and the no-action-effect condition (lower row). The two squares in the first picture depict the areas of interest (AOIs) used for data analysis. The squares were not visible during the experiment.

Participants were presented with 12 repetitions of a video showing how a claw approached and interacted with a toy. In both experimental conditions, the first part of the video was identical: The videos showed the surface of a gray table filmed from the side in front of a gray background with a toy sitting on the table at screen center (see Figure 1). After approximately 1,000 ms, a claw that was painted with a light color entered the scene from the right side of the screen, approached the toy on a linear path, and grasped it (duration approx. 2,140 ms). In the action-effect condition, the claw then lifted the toy up, accompanied by a sound, and put the toy back on the table (duration approx. 2000 ms). Based on prior research, the addition of the sound was not expected to influence infants’ predictive gaze behavior (Adam et al., 2017). Then, the screen froze and the scene was presented for another 3,870 ms until the video ended. In the no-action-effect condition, immediately after the claw grasped the toy, the screen froze for approximately 5,870 ms until the video ended. Thus, both videos were identical in length and had a total running time of about 9,010 ms. Attention-getter videos (e.g., a bouncing ball or a waving hand) were presented in between stimulus videos in order to redirect the participants’ gaze to the screen.

Gaze behavior was recorded with an SMI RED 250 mobile eye tracker mounted to a 22-inch screen. The sampling rate was 250 Hz, and the screen resolution was 1,680 by 1,050 pixels. During the experiment, participants sat on their caregiver’s laps in front of the screen, approximately 60 cm away from the eye tracker. Caregivers had no prior knowledge about the contents of the stimuli or the purpose of the study and were instructed to only interact with their child in case she needed soothing. The experiment started with a 5-point calibration and with manual point acceptance. The calibration stimulus was an animated picture of a pulsating circle in front of a gray background. After successful calibration, the experiment started with a total runtime of about 2.5 min.

### Data Handling

In both conditions, we used the same areas of interest (AOIs) to analyze participants’ gaze behavior (see Falck-Ytter et al., 2006; Kanakogi and Itakura, 2011; Adam et al., 2016, for similar criteria): a static AOI for the goal object and a moving AOI for the claw. Gaze-arrival times were calculated by subtracting the time when participants first fixated the goal AOI from the time when the claw entered the goal AOI. Gaze-arrival times above the value of 0 ms were considered predictive, gaze-arrival times around 0 ms were considered tracking, and gaze-arrival times below 0 ms were considered reactive. A trial was valid when participants first fixated the claw AOI for at least 200 ms before they fixated the goal AOI. Using this criterion of 200 ms (see Gredebäck and Melinder, 2010; Kanakogi and Itakura, 2011; Henrichs et al., 2012; Adam et al., 2016, for a similar use of this criterion) ensured first, that the infants had indeed at least shortly attended to the moving agent and therefore to the movement part of the action. Second, it ensured that infants who just looked at the goal object throughout the action were not included in the analyses, because these sticky fixations would not tell us whether the infant in that particular trial had been predictive. Additionally, values of −1,000 ms or below were classified as invalid. The first trial was excluded from our analyses, because the experimental manipulation of the action effect only occurred at the end of the first video. Participants needed to have at least two valid trials among the analyzed trials 2–12 to be included in our analyses, a criterion that has been used in studies with infants around 6 months of age (Kanakogi and Itakura, 2011; Gredebäck et al., 2018; Adam and Elsner, 2020). The gaze-arrival times in the valid trials were then averaged for every participant to create a mean gaze-arrival time. On average, the 7-month-olds contributed significantly more trials in the action-effect condition (*n =* 7.8, *SD =* 3.4) than in the no-action-effect condition (*n =* 5.1, *SD =* 2.6), *t*(40) = −2.92, *p <* 0.01, *r =* 0.4. Both the 11-month-olds (action-effect condition: *n =* 8.4, *SD =* 3.1; no-action-effect condition: *n =* 8.2, *SD =* 2.6; *t*(39) = −0.23, *p =* 0.82, *r =* 0.04) and the 18-month-olds (action-effect condition: *n =* 9.2, *SD =* 2.1; no-action-effect condition: *n =* 9.4, *SD =* 2.1; *t*(39) = 0.31, *p =* 0.76, *r =* 0.05) contributed a similar number of valid trials in both conditions. In neither age group, there was a significant correlation between the number of valid trials and the mean gaze-arrival time, all *ps >* 0.28.

To test our hypotheses, we conducted ANOVAs and Bonferroni-corrected independent-samples *t*-tests in order to compare the mean gaze-arrival times as a function of the between-subjects factors age group and condition. We also performed one-sample *t*-tests against the threshold of 0 ms for every subgroup to classify the gaze behavior as predictive, tracking, or reactive. In case of a null result, we also included *BF*_01_, indicating the Bayes factor in favor of the H0 over H1 with values between 0 and 3 representing anecdotal evidence, between 3 and 10 representing moderate evidence, and >10 representing strong evidence. Additionally, we used exploratory regression analyses with linear, logarithmic, and quadratic curve fitting in every subgroup to investigate potential changes of the mean gaze-arrival times across trials 2 to 12. When one of the functions for the gaze-arrival times yielded a significant fit, we also performed exploratory regression analyses with the same functions on infants’ fixation times on the claw AOI and on the goal AOI. Linear, logarithmic, and quadratic curve fitting was chosen because significant fits for these types of curves have commonly been reported in prior research (e.g., Henrichs et al., 2012; Adam et al., 2017; Adam and Elsner, 2020).

## Results

**Figure 2:**
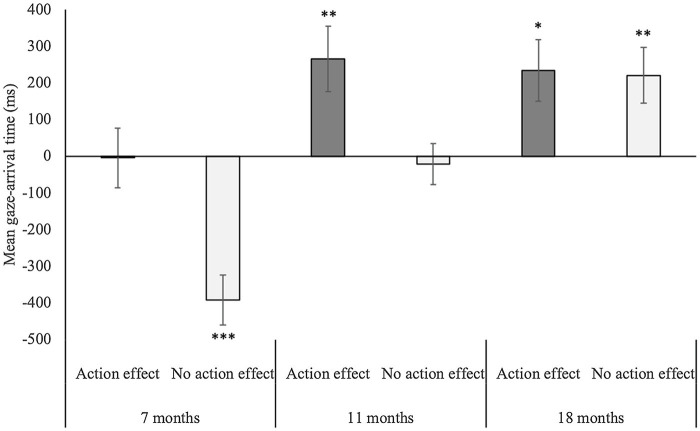
Mean gaze-arrival times for the 7-, 11-, and 18-month-olds in the action-effect and the no-action-effect condition. Positive and negative values represent mean gaze-arrival times before and after the claw arrived at the goal AOI. Error bars represent standard-errors, and the asterisks mark mean gaze-arrival times significantly different from 0 ms. ^*^ = *p* < 0.05, ^**^ = *p* < 0.01, and ^***^ = *p* < 0.001.

The ANOVA on mean gaze-arrival time with age group (7 months vs. 11 months vs. 18 months) and condition (action effect vs. no action effect) as between-subjects factors yielded a significant main effect of age group, *F*(2,118) = 17.0, *p <* 0.001, η^2^ =0.22, a significant main effect of condition, *F*(1,118) = 13.5, *p <* 0.001, η^2^ =0.10, and a significant interaction between age group and condition, *F*(2,118) = 3.2, *p <* 0.05, η^2^ =0.05 (see Figure 2). Regarding the main effect of the age group, post-hoc independent-samples *t*-tests (Bonferroni corrected with α = 0.016) revealed that mean gaze-arrival times did not differ between the 11- and 18-month-olds, *t*(80) = −1.36, *p =* 0.18, *r =* 0.2. However, both the 11-month-olds, *t*(81) = −3.83, *p <* 0.001, *r =* 0.4, and the 18-month-olds, *t*(81) = −5.16, *p <* 0.001, *r =* 0.5, had significantly faster mean gaze-arrival times than the 7-month-olds. The main effect of condition resulted from faster mean gaze-arrival times in the action-effect than no-action-effect condition. To explore the significant interaction, we compared the mean gaze-arrival times between conditions for each age group separately by independent-samples *t*-tests (Bonferroni corrected with α = 0.016). Significantly faster mean gaze-arrival times in the action-effect than no-action-effect condition occurred in both the 7-month-olds, *t*(40) = −3.65, *p <* 0.001, *r =* 0.5, and the 11-month-olds, *t*(39) = −2.74, *p <* 0.01, *r =* 0.4, but not in the 18-month-olds, *t*(39) = −0.12, *p =* 0.91, *r =* 0.02.

The one-sample *t*-tests against the threshold of 0 ms confirmed our expectations: The 7-month-olds’ gaze behavior was reactive in the no-action-effect condition, *t*(20) = −5.74, *p <* 0.001, *r =* 0.8, and tracking in the action-effect condition, *t*(20) = −0.05, *p =* 0.96, *r =* 0.01, *BF*_01_ *=* 6. The 11-month-olds were tracking in the no-action-effect condition, *t*(20) = −0.37, *p =* 0.72, *r =* 0.08, *BF*_01_ *=* 5.6, and predictive in the action-effect condition *t*(19) = 2.96, *p <* 0.01, *r =* 0.6. Finally, the 18-month-olds were predictive in both the no-action-effect condition, *t*(21) = 2.9, *p <* 0.01, *r =* 0.5, and the action-effect condition, *t*(18) = 2.8, *p <* 0.05, *r =* 0.6.

**Figure 3:**
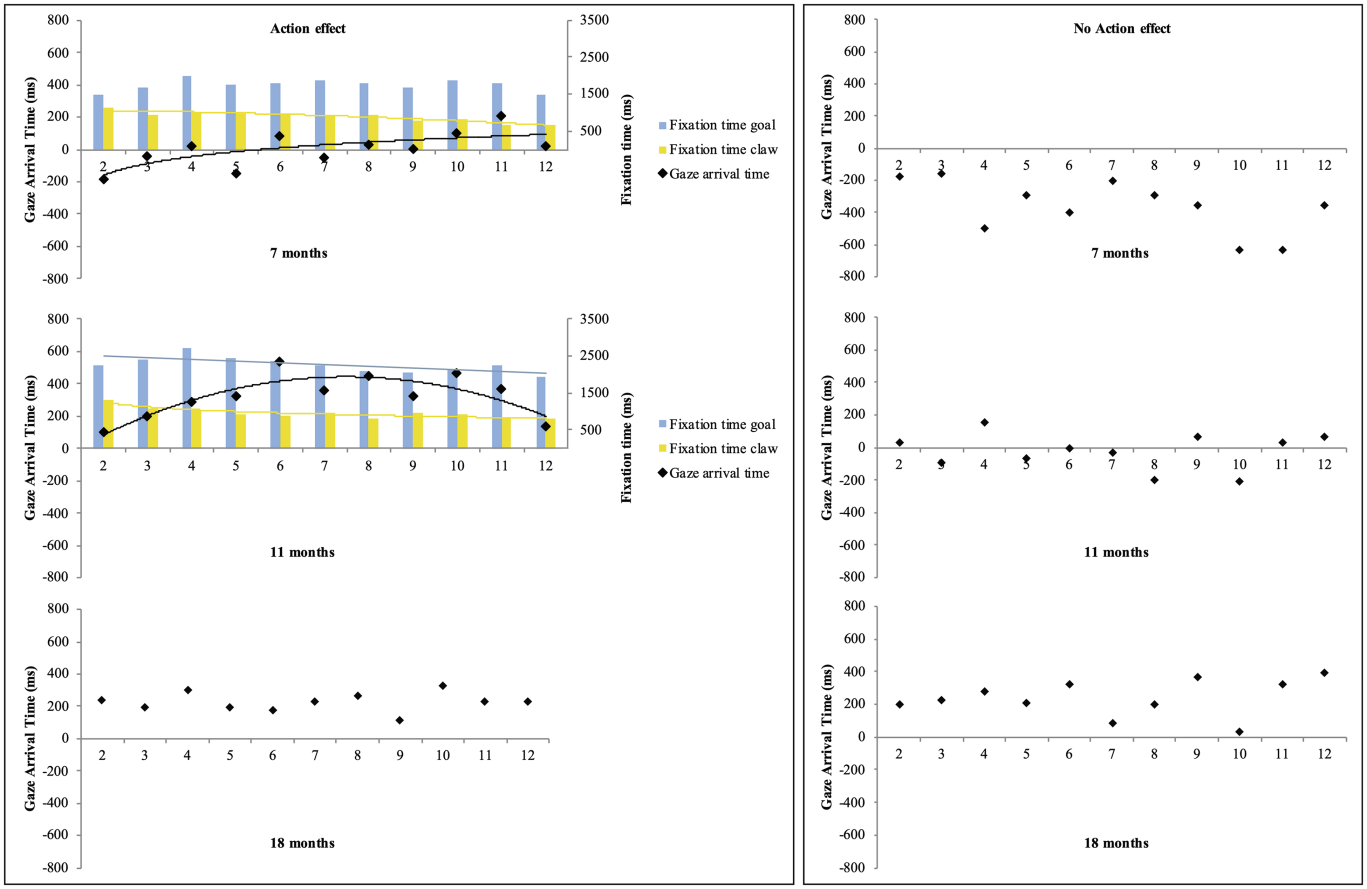
Mean gaze-arrival times (black dots) and fixation times on the goal AOI (blue bars) and the claw AOI (yellow bars) across trials 2–12 for the 7-, 11-, and 18-month-olds in the action-effect and no-action-effect condition. Positive and negative values represent mean gaze-arrival times before and after the claw arrived at the goal AOI. The curves represent the significant fit for the regression functions (linear, logarithmic, or quadratic) with most explained variance.

Regarding potential learning effects, the exploratory regression analyses on mean gaze-arrival times across trials 2–12 in the action-effect condition revealed a significant fit for a logarithmic function for the 7-month-olds (*y =* 137.25ln(x) − 241.55, *R*^2^ *adj =* 0.44, *F*(1,9) = 8.97, *p <* 0.05) and a significant fit for a quadratic function for the 11-month-olds (*y =* 227.96 + 179.37x − 11.98x^2^, *R*^2^adj = 0.66, *F*(2,8) = 10.85, *p <* 0.01). In all other conditions and age groups, the analyses did not yield significant fits, all *ps >* 0.06 (see Figure 3). These results indicate that in the action-effect condition, the mean gaze-arrival times of the 7-month-olds got rapidly faster across the first trials, albeit still with mean gaze-arrival times below or at 0 ms, and the mean gaze-arrival times of the 11-month-olds got faster across the first half of the trials, but then slightly decelerated toward the end, always staying above 0 ms. The 18-month-olds’ gaze-arrival times generally stayed in the predictive value range above 0 ms across trials in both conditions. Additional exploratory regression analyses on 7- and 11-month-olds’ fixation times on the claw AOI and the goal AOI across trials 2–12 in the action-effect condition yielded a significant fit for a quadratic function for the 7-month-olds’ fixation times on the claw (*y =* 1068.06 –10x – 2.46x^2^, *R*^2^*adj =* 0.86, *F*(2,8) = 32.24, *p <* 0.001), as well as a significant fit for a logarithmic function for the 11-month-olds’ fixation times on the claw (*y =* −179.74ln(x) + 1250.84, *R*^2^*adj =* 0.78, *F*(1,9) = 35.39, *p <* 0.001), and a significant fit for a linear function for the 11-month-olds’ fixation times on the goal (*y =* 2530.74 – 44.92x, *R*^2^*adj =* 0.42, *F*(1,9) = 8.34, *p <* 0.05). These results show that across trials, the 7-month-olds in the action-effect condition looked less at the claw, and the 11-month-olds in the action-effect condition looked less at the claw and at the goal object (see Figure 3. For information on fixation times across trials for all six groups, see Supplementary Figure 1).

## Discussion

The aim of the present study was to investigate the impact of the agency cue of producing a salient action effect in interplay with the developing agentive self on 7-, 11-, and 18-month-olds’ goal-predictive gaze shifts during the observation of a non-human grasping action. We investigated at which age and in which conditions infants would be able to produce predictive gaze behavior, and we also looked at potential learning effects across trials. Fitting to our expectations, we found no predictive gaze behavior regardless of the salient action effect in the 7-month-olds, predictive gaze behavior when the salient action effect was presented, but tracking gaze behavior when the salient action effect was not presented in the 11-month-olds, and predictive gaze behavior regardless of the salient action effect in the 18-month-olds. This result pattern replicates previous findings in the context of human actions for mechanical actions and shows how similar patterns show up later during development for non-human compared to human actions, which fits the slightly later occurring developmental milestone of tool use compared to grasping (Adam and Elsner, 2020). Additionally, regarding gaze behavior across trials, in the action-effect condition, we found a significant fit for a logarithmic function for the 7-month-olds and a significant for a quadratic function for the 11-month-olds, indicating systematic changes of gaze behavior across trials: The 7-month-olds showed increasing mean gaze-arrival times across the course of the experiment, whereas the 11-month-olds showed increasing mean gaze-arrival times in the first half of the experiment, but decreasing mean gaze-arrival times in the second half (while still staying in the predictive value range).

First, our findings from the 7-month-olds replicate prior research by showing that even in the presence of a salient action effect, infants at this age do not use this information in order to predict the goal of the mechanical claw (Adam and Elsner, 2020). Additionally, the present study expands these findings by showing that although infants’ gaze behavior on average was not predictive in the action-effect condition, mean gaze-arrival times in this condition were still significantly faster (i.e., classified as tracking) than in the no-action-effect condition (i.e., classified as reactive). This shows that the agency cue had an impact on the 7-month-olds’ gaze behavior, but that ultimately, infants still did not arrive with their gaze at the goal object ahead of time. Further, regression analyses revealed that the 7-month-olds in the action-effect condition showed rapidly increasing mean gaze-arrival times across the first trials. This implies that observing the action effect might have triggered the 7-month-olds’ (still weak) action knowledge to a certain degree, and that across trials, the infants indeed used the bottom-up information in the form of the action effect to produce faster gaze shifts toward the end of the experiment. It is interesting to note that the 7-month-olds’ gaze behavior for the grasping claw in the action-effect condition was more comparable to the gaze behavior of 6-month-olds, not 7-month-olds, for a grasping hand exhibiting an action effect (Elsner and Adam, 2020), and that in the present no-action-effect condition, the 7-month-olds’ mean gaze-arrival times were even lower than that of the 6-month-olds for the grasping hand without an action effect. These results fit the idea that due to the later developing developmental milestone of tool-use compared to grasping (e.g., McCarty et al., 2001), the internal models for mechanical claws also develop later than the internal models for human hands. Therefore, based on the assumptions of the CAPRI model (Gumbsch et al., 2021) and Elsner and Adam (2020), we conclude that the 7-month-olds did not yet have strong action-event schemata for actions performed by mechanical claws, which resulted in tracking gaze behavior or in the case of the no-action-effect condition, even reactive gaze behavior, to maximize information gain and to minimize uncertainty by closely observing the agent and its movement. However, it is possible that the 7-month-olds would be able to predict the claw’s action goal when more agency cues were provided, for example, when the claw moved biologically or was self-propelled (e.g., Premack, 1990; Baron-Cohen, 1994; Bíró and Leslie, 2007).

Second, the present findings replicated that 11-month-olds show predictive gaze behavior when a mechanical claw displays agency cues, such as the production of a salient action effect (Adam et al., 2017; Adam and Elsner, 2018). Additionally, regression analyses revealed that for the 11-month-olds, the observation of the action effect resulted in increasing mean gaze-arrival times (in an overall predictive value range) across the first half of the experiment and in a slight decrease (though still in the predictive value range) across the second half. In contrast, in the no-action-effect condition, the 11-month-olds’ gaze followed the claw to the goal, with no systematic change across trials. These results are in line with our expectations, confirming that seeing the grasping claw and action effect probably triggered 11-month-olds’ grasping experience as well as their emerging action knowledge about tool use (e.g., McCarty et al., 2001). Furthermore, the 11-month-olds’ mean gaze-arrival times in both conditions were strikingly similar to the ones found by Adam and Elsner (2020) at 7 months for a grasping human hand. This further indicates that, when stored action-event schemata are still relatively weak, infants can benefit from the display of agency cues, because the cues exert stronger activation of the stored action-event schemata and a stronger agency attribution to the claw (at 11 months). This activation, in turn, may enable goal prediction for subsequent observations of this action event *via* forward modeling and top-down processes (Elsner and Adam, 2020; Gumbsch et al., 2021).

Third, the findings from the 18-month-olds revealed predictive gaze behavior in both conditions. Thus, this study is the first to show that infants at 18 months are able to predict the goal of an ongoing grasping action of a mechanical claw regardless of the salient action effect, that is, in the absence of any additional bottom-up information. Additionally, regression analyses revealed no systematic change of gaze behavior across trials, because the 18-month-olds already started out with predictive mean gaze-arrival times in the first trials. These results indicate that 18-month-olds had already built up strong internal models and strong action-event schemata with regard to grasping and tool-use actions, which they could use as top-down information during the initial observation of the claw, the potential goal object, and the start of the goal approach. At 18 months of age, infants are already quite apt at simple tool-use actions to retrieve a distant object (starting around 14 months of age; McCarty et al., 2001). Based on this, we would possibly find similar results already at an earlier age. However, we chose to study 18-month-olds because infants’ ability to produce an action does not instantly guarantee that they would also be able to predict the goal during observation of this action (e.g., Gredebäck et al., 2018; Adam and Elsner, 2020).

The results from the regression analyses for the 7- and 11-month-olds do not match prior findings in which no learning effects were reported for 7-month-olds (Adam and Elsner, 2020) and in which 11-month-olds were reported to show rapidly faster mean gaze-arrival times in the predictive value range across the first trials, but no decreasing mean gaze-arrival times in the second half of the experiment (Adam et al., 2017). Here, it needs to be noted that infants’ limited attention span allows for only a very limited number of trials, providing only a weak basis for the analysis of learning effects. For example, the regression analyses on 7- and 11-month-olds’ fixation times to the claw AOI and the goal AOI in the action-effect condition revealed that across trials, the 7-month-olds looked less at the claw, and the 11-month-olds looked less at both the claw and the goal object, indicating a decreasing interest in the presented stimuli over the course of the experiment. These results fit to prior research indicating that infants’ looking times tend to decrease when a stimulus is repeated multiple times (e.g., Woodward, 1998, 1999). Therefore, it does not come as a surprise that in studies on infants goal-predictive gaze behavior, the results on learning effects across trials are generally unstable and seem to occur unsystematically, even when they are measured with similar stimuli (e.g., Henrichs et al., 2012). Therefore, interpretations of these findings have to be made with caution, and further systematic research on the factors driving learning effects during action observation is needed. For example, it remains unclear whether there is a systematic relation between fixation times on the stimulus display across trials and the corresponding mean gaze-arrival times.

Admittedly, the present findings are ambiguous about whether infants’ gaze behavior directly relied on infants’ experience with or knowledge about the observed action, or on general cognitive maturation. The role of general maturation processes seems to be supported by the fact that infants’ general ability to disengage their gaze from an interesting stimulus improves across the first year of life (Elsabbagh et al., 2013). However, predictive gaze behavior differed as a function of producing a salient action effect in 7-month-olds for a human hand, and in 11-month-olds for a mechanical claw (Adam and Elsner, 2020), which cannot be explained solely by general cognitive maturation processes. Training studies in which infants learn to perform novel actions could be used to disentangle these factors by investigating the impact of systematic manipulation of such learning experience on predictive gaze behavior. For example, a short training session in which infants were encouraged to actively engage in a novel action altered infants’ subsequent looking times during observation of that action, indicating changed attribution of goal-directedness (Sommerville et al., 2005; Woodward, 2009; Gerson and Woodward, 2014b). However, the effect of training sessions on infants‘ predictive gaze behavior still needs to be investigated in more detail.

An alternative explanation of our results across the age groups could be that the increasing gaze-arrival times merely reflect the increasing size of infants’ functional visual field (e.g., Hullemann and Olivers, 2017). Based on this idea, older (but not younger) infants could have detected the goal object *via* peripheral vision, which in turn triggered an early gaze shift, without any involvement of action processing or the activation of action-event schemata. However, this cannot explain why same-aged infants (i.e., the 7- and 11-month-olds), with functional visual fields matured to a certain size, exhibited significantly higher gaze-arrival times in the action-effect-condition than in the no action-effect-condition. Additionally, Adam and Elsner (2020) found the same pattern of gaze behavior for observations of a grasping hand in younger age groups with a comparably less developed functional visual field. Therefore, although general maturation processes regarding infants’ cognition may play a role, action-related cognitive processing has to be in place in order to fully account for our findings.

Another alternative interpretation may be that infants’ predictive gaze behavior is not specific to the observation of goal-directed actions performed by agents, but is instead elicited by associative learning of the objects’ movements. That is, infants may have shifted their gaze to the goal object because they had learned that “when object A (claw) touches object B (goal object), object B will start moving”. For the action-effect condition, we cannot fully exclude impacts of such general learning mechanisms of simple associations between moving objects. However, these mechanisms fail to explain why infants’ predictive gaze behavior varies with the familiarity of the observed agent (e.g., Falck-Ytter et al., 2006; Kanakogi and Itakura, 2011; Cannon and Woodward, 2012; Adam et al., 2016). For example, 7-month-olds showed predictive gaze shifts for an effect-producing grasping human hand (Adam and Elsner, 2020), but in the present study did not predict the goal of an almost identical action of a claw. In addition, infants’ predictive gaze behavior depends on specific features of the “agent” and of the movement, in particular on cues that signal agency (e.g., Bíró, 2013). Therefore, we take our findings to reflect infants’ cognitive processing of observed actions rather than simple associative learning of regularities in the movements of random objects.

Taken together, our results expand the previous work on infants’ goal-predictive gaze behavior in the context of human hands to simple actions performed by a non-human agent. Framed according to the theoretical model by Elsner and Adam (2020) and the CAPRI model (Gumbsch et al., 2021), at 7 months, infants’ stored action representations are probably still too weak to enable predictive gaze behavior, even in the presence of the agency cue of producing a salient action effect (Bíró and Leslie, 2007). At 11 months of age, infants’ stored action representations are still weak, but strong enough to be activated by some observations of the production of a salient action effect during the first trials, which enables goal prediction upon observing the action’s start in subsequent trials. Finally, at 18 months, infants’ stored action representations are strong enough to be activated already for the first action observations, even in the absence of any additional agency cues. Therefore, these results provide further evidence for the role of the developing agentive self and the interplay between bottom-up and top-down information during the observation of goal-directed actions and illustrate how the shifted developmental courses of this behavior follow infants’ motor development and acquired action experience with mechanical agents compared to human agents (Csibra, 2007; Southgate, 2013; Elsner and Adam, 2020; Gumbsch et al., 2021). Future research should further investigate the specific role of infants’ agentive experience with the observed action by applying training paradigms, which would shed more light on the interplay of perceptual bottom-up information and experience-based top-down processes underlying the developmental course of infants’ goal-predictive gaze behavior.

## Data Availability Statement

The raw data supporting the conclusions of this article will be made available by the authors, without undue reservation.

## Ethics Statement

The studies involving human participants were reviewed and approved by the Ethics Committee of the University of Potsdam. Written informed consent to participate in this study was provided by the participants’ legal guardian/next of kin.

## Author Contributions

MA wrote the manuscript. All authors were involved in the design of the study, data collection, data analyses, and contributed to the manuscript intellectually.

## Conflict of Interest

The authors declare that the research was conducted in the absence of any commercial or financial relationships that could be construed as a potential conflict of interest.

## Publisher’s Note

All claims expressed in this article are solely those of the authors and do not necessarily represent those of their affiliated organizations, or those of the publisher, the editors and the reviewers. Any product that may be evaluated in this article, or claim that may be made by its manufacturer, is not guaranteed or endorsed by the publisher.
