# Supplementary figures and images for: The Impact of Action Effects on Infants’ Predictive Gaze Shifts for a Non-Human Grasping Action at 7, 11, and 18 Months
Source: Front Psychol. 2021 Aug 10;12:695550. doi: 10.3389/fpsyg.2021.695550 (PMC8382717; doi:10.3389/fpsyg.2021.695550)

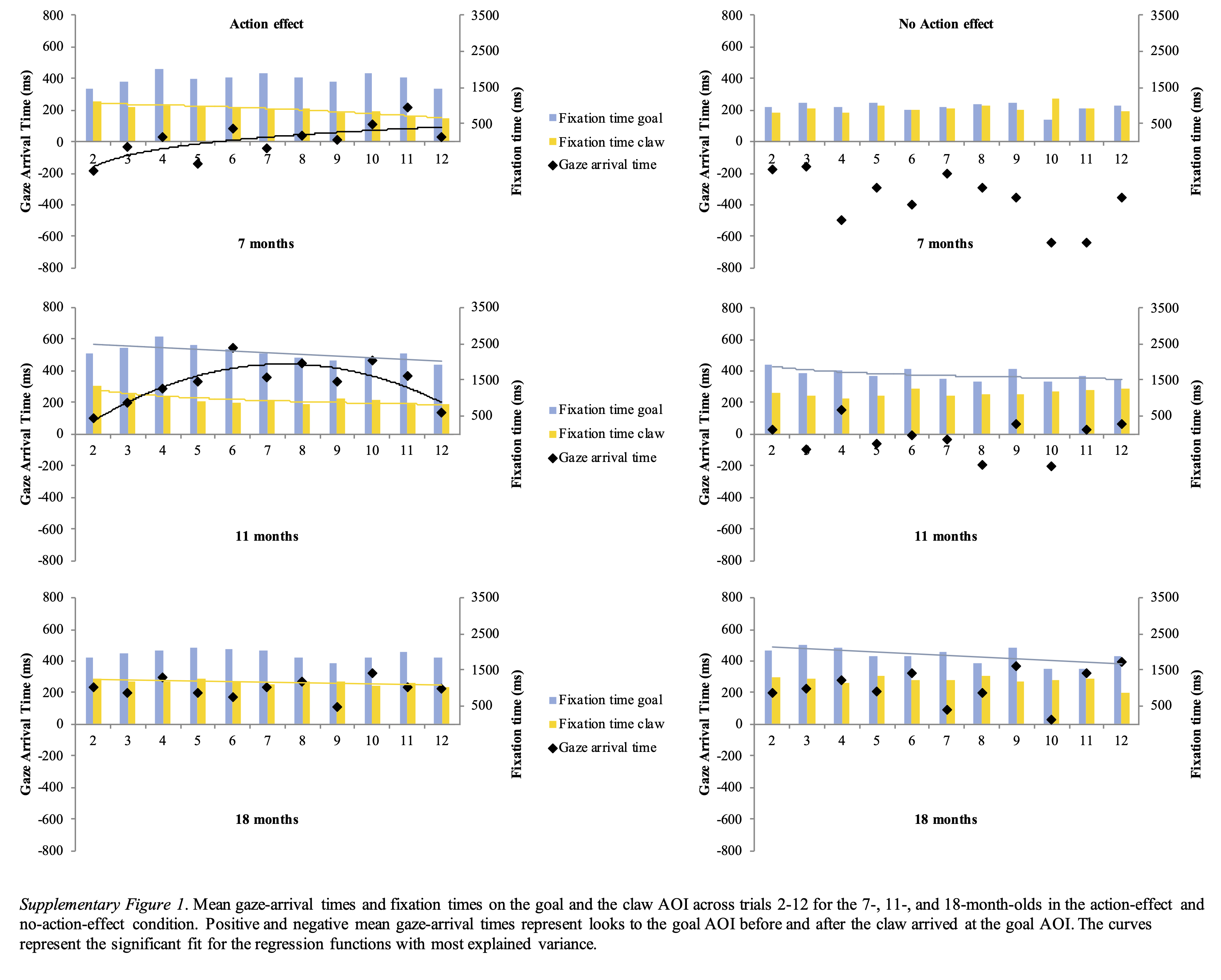

Supplement: Supplementary file 1 [file Image_1.TIFF]
